# The Role of Operative Laparoscopy in Gynecologic Oncology

**DOI:** 10.1155/DTE.2.185

**Published:** 1996

**Authors:** E. M. Hartenbach, J. M. Fowler

**Affiliations:** 1 Women's Cancer Center Department of Obstetrics and Gynecology University of Minnesota Hospital and Clinics Minneapolis Minnesota USA; 2 Division of Gynecologic Oncology Box 395 Mayo Memorial Building 420 Delaware Street Southeast Minneapolis MN 55455 USA

## Abstract

The potential applications of operative laparoscopy have expanded with improvements in technology and instrumentation. With newly developed techniques to complete both pelvic and paraaortic lymph node dissection, the use of the laparoscope has increased in patients with pelvic malignancies. Gynecologic oncologists are currently incorporating the techniques of operative laparoscopy in the management of patients with cervical, endometrial, and ovarian cancer. Multicenter prospective clinical trials are necessary to further define the role of laparoscopy in gynecologic oncology.


*Diagnostic and Therapeutic Endoscopy*, 1996, Vol. 2, pp. 185–188
Reprints available directly from the publisher
Photocopying permitted by license only

© 1996 OPA (Overseas Publishers Association)
Amsterdam B. V. Published in The Netherlands
by Harwood Academic Publishers GmbH
Printed in Singapore

### The Role of Operative Laparoscopy in Gynecologic Oncology

E. M. HARTENBACH and J. M. FOWLER

*Women's Cancer Center, Department of Obstetrics and Gynecology, University of Minnesota Hospital and Clinics, Minneapolis, Minnesota*

*(Received July 19, 1995; in final form November 27, 1995)*

The potential applications of operative laparoscopy have expanded with improvements in technology and instrumentation. With newly developed techniques to complete both pelvic and paraaortic lymph node dissection, the use of the laparoscope has increased in patients with pelvic malignancies. Gynecologic oncologists are currently incorporating the techniques of operative laparoscopy in the management of patients with cervical, endometrial, and ovarian cancer. Multicenter prospective clinical trials are necessary to further define the role of laparoscopy in gynecologic oncology.

**KEY WORDS:** Laparoscopy, gynecologic malignancies, laparoscopic lymphadenectomy, laparoscopic surgical staging

Historically, the laparoscope has been used for diagnostic purposes and for sterilization procedures. Due to technologic advances, operative laparoscopy now plays a role in the management of a wide variety of benign gynecological conditions including ectopic pregnancy, endometriosis, pelvic pain, leiomyomata, and adnexal masses. Similarly, the role of operative laparoscopy in the management of malignant disease has expanded. With newly developed techniques to complete both pelvic and paraaortic lymph node dissection, the use of the laparoscope has increased in patients with pelvic malignancies. Gynecologic oncologists are currently incorporating the techniques of operative laparoscopy in the management of patients with cervical, endometrial, and ovarian cancer.

The main role for operative laparoscopy in women with gynecologic neoplasms is in surgical staging. The technique of laparoscopic pelvic lymph node dissection was first described in women with cervical carcinoma by Querleu et al. in 1991 (1). Subsequently, other investigators have reported on the safety and efficacy of this procedure in patients with cancers of the cervix, endometrium, and ovary (2–4). Critical to the addition of laparoscopy to oncology was the development of techniques for paraaortic lymphadenectomy. For surgical staging to be complete, access to bilateral paraaortic nodal tissue is imperative (Fig. 1). Nezhat et al. (5) first published a case report of a laparoscopic radical hysterectomy that included a low right paraaortic nodal dissection in 1992. Others had previously begun to develop strategies for laparoscopic paraaortic lymph node sampling in animal models (6). Currently, there are a number of series that confirm the feasibility of removing bilateral paraaortic lymph nodes laparoscopically (2,4,7).

The most significant experience to date with laparoscopic lymphadenectomy is in women with cervical carcinoma. Cervical carcinoma remains a clinically staged disease as recommended by the International Federation of Gynecology and Obstetrics (FIGO). However, clinical staging is inadequate in estimating tumor spread. The discrepancy between clinical and surgical staging ranges from 20 to 48% depending on the clinical stage (8). The primary benefit of surgical staging is the ability to identify patients who may benefit from extended field irradiation. Although the value of surgical staging remains controversial, management decisions based on the information result in an improvement in survival in 2.5 to 7% of women whose cancer is surgically staged (8,9). Other advantages of surgical staging include the removal of bulky lymph nodes, removal of diseased adnexae, and ovarian transposition.

Address for correspondence: Jeffrey M. Fowler, M.D., Division of Gynecologic Oncology, Box 395, Mayo Memorial Building, 420 Delaware Street Southeast, Minneapolis, MN 55455. Tel: 612-626-4338; 612-626-0665.

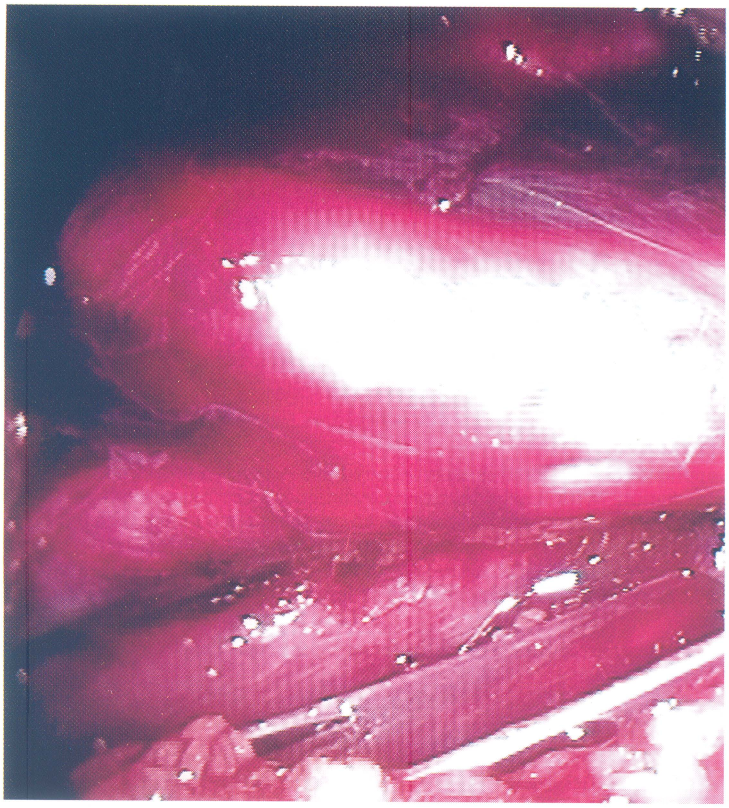

**Figure 1** Left-sided paraaortic lymph node dissection accomplished via laparoscopy.

Laparoscopic lymphadenectomy in cervical cancer patients is utilized in two clinical settings (10). In patients with early stage disease (FIGO IA–IIA) that is suitable for radical hysterectomy, lymph node sampling is carried out before radical hysterectomy. Lymph nodes are assessed by frozen section and radical hysterectomy is performed if they are negative for malignancy. In patients with FIGO IB–IIA disease as many as 5 to 10% will have positive common iliac and paraaortic lymph nodes (11). Therefore, laparoscopic sampling can save these patients from undergoing a laparotomy. If the lymph nodes are positive, the surgical staging is completed laparoscopically, and the patient is then treated with radiation therapy. In contrast, patients with advanced disease (FIGO IIB–IV) are surgically staged with laparoscopic pelvic and paraaortic lymphadenectomy. Removal of bulky, grossly positive lymph nodes can be carried out in the majority of these patients. Lymph node yield is adequate and compares with that of staging laparotomies (3,10). Radiation can then be tailored to disease extent. There is some concern that transperitoneal laparoscopic lymphadenectomy techniques may increase radiation-related enteric morbidity. However, animal studies indicate that transperitoneal laparoscopic procedures may not produce any more surgical adhesions than extraperitoneal procedures (12). In addition, to the use of the laparoscope for surgical staging, there are case reports of laparoscopic radical hysterectomy and laparoscopically assisted radical vaginal hysterectomy (5,13,14). It remains to be seen whether these procedures will be incorporated into current clinical practice.

Operative laparoscopy is also useful in the management of patients with malignancies of the uterine corpus. In 1988, endometrial cancer became a surgically staged malignancy according to FIGO. The importance of pelvic and paraaortic lymph node status documented by a large Gynecologic Oncology Group (GOG) study was instrumental in motivating the change to surgical staging (15). In 1992, Childers and Surwit (16) reported the use of a combined laparoscopic and vaginal approach in the management of two cases of stage I adenocarcinoma of the endometrium. The lymph node dissection, mobilization of the adnexae, and procurement of peritoneal cytology were performed laparoscopically and then the uterus, tubes, and ovaries were removed vaginally. The subsequent larger series published by Childers et al. (2) documented that the approach is feasible and has an acceptable complication rate. The main advantage is a shortened hospital stay and recovery time. A recent abstract presented data on a comparison between laparoscopic management and traditional management in patients with endometrial cancer (17). Patients managed with a laparoscopic approach had the same number of lymph nodes removed, but had less complications, a shorter hospital stay, and quicker recovery than the laparotomy group.

In addition to surgical staging in the primary management of endometrial carcinoma patients, the technique can be utilized in patients with incomplete staging of disease at their primary surgery. Childers et al. (18) reported on 13 of these patients with presumed stage I disease referred after hysterectomy. Laparoscopic lymphadenectomy was completed in all patients and residual disease was noted in three. Laparoscopic restaging is an alternative for patients referred after incomplete staging of a variety of malignancies including uterine sarcomas, epithelial ovarian carcinomas, sex cord stromal ovarian tumors, and germ cell tumors of the ovary.

The use of laparoscopy or peritoneoscopy in the management of epithelial ovarian cancer was first described in 1973 by Bagley et al. (19). The laparoscope was used to evaluate patients before and after a chemotherapy protocol. This initial report highlighted the ability of the laparoscope to visualize subdiaphragmatic metastasis likely to be undetected by conventional laparotomy. Subsequent studies confirmed that laparoscopy can be used for disease assessment or "second-look" surgery (20,21). However, in 1981, Ozols et al. (21) reported that in patients with a negative second look laparoscopy, immediate laparotomy revealed residual disease in 55%. Many gynecologic oncologists concluded that a laparotomy was required in all cases to confirm the absence of disease. These results were reported before modern advances in laparoscopic surgery, and many laparoscopic surgeons now believe that second-look procedures can be carried out with the same false-negative rate as laparotomy. Current techniques of adhesiolysis and lymphadenectomy contribute to disease reassessment surgery in these patients. Childers et al. (22) recently reported on 44 laparoscopic second-look procedures with complication rates similar to surgery via laparotomy. Similarly, the accuracy in detecting disease (56%) compared favorably with laparotomy.

As mentioned above, another group of patients with ovarian malignancies that have benefited from laparoscopic advances are those patients referred after incomplete staging. Patients who have undergone a total abdominal hysterectomy and bilateral salpingo-oophorectomy without lymphadenectomy can have a laparoscopic pelvic and paraaortic lymphadenectomy performed. This is particularly important for early-stage disease, for which as many as 22% of stage I malignancies are upstaged based on lymph node involvement.

Querleu et al. (23) performed laparoscopic restaging on nine patients with apparent early-stage carcinoma of the ovary or fallopian tube, but with incomplete staging during a previous surgical procedure. The authors were able to complete staging in all nine patients including infrarenal, paraaortic lymph node dissections. They reported no major complications related to the procedure. Alternatively, patients can be treated with chemotherapy, and then a second-look laparoscopy procedure with lymphadenectomy can be performed for reassessment and surgical staging.

It is clear that laparoscopic approaches to the management of gynecologic malignancies are feasible and provide exciting alternatives. However, the safety and efficacy of operative laparoscopy compared to laparotomy in this setting has not been carefully studied. Potential advantages include shorter operative time for some procedures, shorter recovery times, and less adhesion formation. These new surgical techniques need to be evaluated critically and compared to more traditional approaches. Currently, the GOG, a multicenter cooperative group, is conducting studies to evaluate the use of operative laparoscopy in the management of cervical, endometrial, and ovarian cancer patients. Multicenter prospective clinical trials like these are necessary to further define the role of laparoscopy in gynecologic oncology.

### REFERENCES

1. Querleu D, LeBlanc E, Castelain B. Laparoscopic pelvic lymphadenectomy in the staging of early carcinoma of the cervix. Am J Obstet Gynecol 1994;164:579.
2. Childers JM, Brzechffa PR, Hatch KD, et al. Laparoscopically-assisted surgical staging (LASS) of endometrial cancer. Gynecol Oncol 1993;51:33.
3. Fowler JM, Carter JR, Carlson JW, et al. Lymph node yield from laparoscopic lymphadenectomy in cervical cancer: a comparative study. Gynecol Oncol 1993;51:187.
4. Childers JM, Hatch KD, Tran A, et al. Laparoscopic paraaortic lymphadenectomy in gynecological malignancies. Obstet Gynecol 1993;82:741.
5. Nezhat CR, Burrell MO, Nezhat FR, et al. Laparoscopic radical hysterectomy with paraaortic and pelvic node dissection. Am J Obstet Gynecol 1992;166:864.
6. Herd J, Fowler JM, Shenson D, et al. Laparoscopic paraaortic lymph node sampling: development of a technique. Gynecol Oncol 1992;44:271.
7. Querleu D. Laparoscopic paraaortic node sampling in gynecology oncology: a preliminary experience. Gynecol Oncol 1993;49:24.
8. LaPolla JP, Schlaerth JB, Gaddis O, et al. Influence of surgical staging on the evaluation and treatment of patients with cervical carcinoma. Gynecol Oncol 1986;24:194.
9. Potish RA, Twiggs LB, Okagaki T, et al. Therapeutic implications of the natural history of advanced cervical cancer as defined by pretreatment surgical staging.

10. Hallum A, Childers J. Laparoscopy in the treatment of early cervical carcinoma diagnostic and therapeutic endoscopy. Diagn Ther Endosc 1994;1:19.
11. Morrow CP. Synopsis of gynecological oncology. In: Morrow CP, Curtin JP, Townsend DE, eds. New York: Churchill Livingstone, 1993:111.
12. Fowler JM, Hartenbach EM, Reynolds HT, et al. Pelvic adhesion formation after pelvic lymphadenectomy: comparison between transperitoneal laparoscopy and extraperitoneal laparotomy in a porcine model. Gynecol Oncol 1994;55:25.
13. Querleu D. Laparoscopically-assisted radical vaginal hysterectomy. Gynecol Oncol 1993;51:248.
14. Dargent D, Roy M, Kelka N, et al. The Schauta operation: its place in the management of cervical cancer in 1993. Abstract presented at the 24th Annual Meeting of The Society of Gynecologic Oncologists, 1993.
15. Creasman WT, Morrow CP, Bundy BN, et al. Surgico pathological spread pattern of endometrial carcinoma: a gynecological oncology group study. Cancer 1987;60:2035.
16. Childers JM, Surwit EA. Combined laparoscopic and vaginal surgery for the management of two cases of stage I endometrial cancer. Gynecol Oncol 1992;45:46.
17. Boike G, Lurain J, Burke J. A comparison of laparoscopic management of endometrial cancer with traditional laparotomy. Gynecol Oncol 1994;52:105.
18. Childers JM, Spiritos NM, Brainard P, et al. Laparoscopic staging of the patient with incompletely staged early adenocarcinoma of the endometrium. Obstet Gynecol 1994;83:597.
19. Bagley CM, Young RC, Schein PS, et al. Ovarian carcinoma metastatic to the diaphragm—frequently undiagnosed at laparoscopy. Am J Obstet Gynecol 1973;110:397.
20. Berek JS, Griffiths CT, Leventhal JM. Laparoscopy for second-look evaluation in ovarian cancer. Obstet Gynecol 1981;58:192.
21. Ozol RF, Fisher RI, Anderson T, et al. Peritoneoscopy in the management of ovarian cancer. Am J Obstet Gynecol 1981;140:611.
22. Childers JM, Lang J, Surwit EA, Hatch KD. Laparoscopic surgical staging of ovarian cancer. Gynecol Oncol, in press.
23. Querleu D, LeBlanc E. Laparoscopic Infrarenal paraaortic lymph node dissection for restaging of carcinoma of the ovary or fallopian tube. Cancer 1994;73:1467.

